# Preferences and heterogeneity in care service needs among disabled older adults in an urban setting

**DOI:** 10.3389/fpubh.2025.1727372

**Published:** 2026-01-19

**Authors:** Xuewei Zhao, Jun Zhao

**Affiliations:** 1 The Fourth Affiliated Hospital of Nanjing Medical University, Teaching and Education Department, Nanjing, China; 2 School of Health Policy and Management, Nanjing Medical University, Nanjing, China; 3 The First Affiliated Hospital of Nanjing Medical University, Hospital Management Research Institution, Nanjing, China

**Keywords:** care service needs, care service preferences, disabled older adults, discrete choice experiment, Willingness-to-pay (WTP)

## Abstract

**Background:**

Against the backdrop of an accelerating aging population, notable disparities exist in the demand for care services among the older adult.

**Objective:**

This study aims to elucidate the heterogeneous preferences and willingness-to-pay for care services among self-caring older adults and those with varying levels of functional disability, in order to provide evidence-based insights for optimizing resource allocation.

**Methods:**

A discrete choice experiment (DCE) was designed, incorporating five core attributes: service content, delivery mode, provider type, cost, and payment mechanism. Using stratified random sampling, 600 self-caring and functionally impaired older adult individuals (Barthel Index ≤ 60 for the functionally impaired) from an urban city were enrolled. After excluding incomplete responses, 579 valid questionnaires were analyzed, comprising 218 self-caring, 231 moderately disabled, and 130 severely disabled older adults. Data analysis was performed using a conditional logit model.

**Results:**

The conditional logit model analysis revealed that the level of disability significantly influenced service preferences. Moderately disabled individuals exhibited significant preferences for service upgrade packages (β = 0.164) and partial reimbursement (β = 0.329), with a willingness-to-pay (WTP) of 149.37 CNY for the upgrade package. In contrast, severely disabled individuals demonstrated significant preferences for institutional care (β = 0.153) and partial reimbursement (β = 0.308), exhibiting a WTP for institutional care as high as 899.56 CNY, which is eight times that of the self-caring group (111.59 CNY). All groups expressed aversion to care provided by nursing aides (β = −0.331 to −0.617), requiring compensation ranging from 322.47 CNY (self-caring) to 1949.40 CNY (severely disabled) to accept such services.

**Conclusion:**

This study demonstrates that the level of disability is a key determinant of care preferences among the older adult, characterized by an evolution in demand from “life support” to “professional medical care.” We recommend establishing a tiered care system: providing a “home-based + upgraded services” model as the core for moderately disabled individuals, and creating a service network dominated by “institutional + professional medical care” for the severely disabled, complemented by a sliding-scale payment mechanism that prioritizes coverage for professional services under long-term care insurance. This approach aims to achieve precise allocation of care resources. The findings provide crucial evidence for the accurate provision of care services in an aging society, contributing to the policy goal of “aging with security.”

## 1 Introduction

The aging population in china is becoming increasingly serious, and older adult care has become a major concern in china. According to the “China older adult Health Report,” the number of disabled people over 60 years old in China will reach 46.54 million from 2021 to 2023; although the disability rate is declining, the number of disabled people is also slowly increasing as the size of the older adult population increases. It is estimated that by 2050, the number of disabled people will reach 52.05 million, accounting for 13.68% of the older adult population (1). As the number of disabled people gradually increases, the perfection of the corresponding service industry becomes particularly important. However, the resource allocation and service capabilities of china's health system and service industry in this regard are still immature (2). In response to this challenge, China has put forward new development requirements for this series of issues in the “14th Five-Year Plan,” such as improving the older adult care service system that coordinates home and community institutions (3) and combines medical care, nursing care and health care (4). However, the implementation of the policy is still subject to practical bottlenecks such as shortage of professional talents and rigid payment mechanism (5).

There are differences in the needs of disabled older adults for nursing services, and there are many unmet needs (6). Research on the real needs of this group of people is still incomplete, and service supply cannot be fully adapted (7). The differences mainly come from the degree of disability, economic level and family support capacity (8). However, the existing service system has not effectively responded to these differentiated needs: the service content is highly homogenized, and community nursing homes generally only provide basic living care; there is a serious shortage of professional nursing staff, and caregivers have not received standardized training (9). The imperfection of the payment mechanism (10) further exacerbates the contradiction. The long-term care insurance pilot has insufficient coverage (11), and the reimbursement scope is mostly limited to institutional services. The reimbursement ratio for home care is insufficient, resulting in a mismatch between supply and demand. Therefore, it is urgent to quantify the preference structure of the disabled population through empirical research to provide a scientific basis for the tiered service system and precise payment policy.

This study focuses on functionally impaired older adult in urban areas, primarily based on the following three considerations: Firstly, urbanization has accelerated demographic shifts, resulting in a large and continuously growing population of urban disabled older adults whose care needs are concentrated and urgent, posing more direct challenges to public resource allocation. Secondly, compared to rural areas, the urban older adult care service system is relatively more mature, with more diverse types of service provision, providing a practical foundation for studying preferences across different service attributes. Thirdly, the urban older adult population exhibits greater heterogeneity (e.g., in income, education level, family structure), facilitating in-depth analysis of factors beyond disability level that influence preferences. The research findings hold stronger guiding value for constructing targeted and stratified urban care service policy systems.

While prior research has documented general trends in older adult care demand, there remains a critical gap in quantitatively assessing the nuanced preferences and willingness-to-pay (WTP) across distinct disability levels. Existing studies often rely on descriptive analyses or simple ranking exercises, which fail to capture the trade-offs older adult individuals are willing to make among multi-attribute care services. This study addresses this gap by employing a Discrete Choice Experiment (DCE) to quantify the relative importance of different care attributes and the heterogeneous WTP across disability strata. The findings thus provide granular, evidence-based insights crucial for moving beyond one-size-fits-all policy approaches.

## 2 Targets and methods

### 2.1 Survey targets

Considering the urban-rural differences and the supply of older adult care services, this study mainly explores the care service preferences of disabled older adults in cities. The study focuses on Nanjing City, collects 600 data in total. A multi-stage, stratified random sampling method was employed. First, eight administrative districts in Nanjing were purposively selected to ensure socioeconomic diversity. Second, within each district, three to five communities were randomly selected using a probability-proportional-to-size sampling approach. Finally, within each community, a simple random sampling method was used to recruit eligible older adult participants from the community registry. Excludes invalid questionnaires, and includes 579 questionnaires for analysis, with an efficiency of 96.5%. Among them, there are 218 people in the self care group, 231 people in the mild disability group and 130 people in the severe disability group. The survey subjects meet the following requirements: disabled older adults aged 60 or above, the respondents are permanent residents of the city, informed and willing to participate; because disabled older adults are often accompanied by dementia, if they cannot complete the questionnaire independently, their family members can answer the questionnaire on their behalf.

Definition of disability level: This questionnaire defines functional limitation of the older adult based on the content of the China Health and Retirement Longitudinal Study (CHARLS) questionnaire (12). All questions asking respondents about activities of daily living (ADL) and instrumental activities of daily living (IADL) have four options: no difficulty, difficulty but can be completed, difficulty and need help, and inability to complete. This paper defines any two options of difficulty but can be completed and difficulty and need help as mild and moderate functional limitation, i.e., mild to moderate disability; inability to complete is defined as severe functional limitation, i.e., severe disability. Based on the Barthel Index classification criteria, the level of disability was categorized as follows: ① Self-care (Barthel Index = 100 points); ② Mild to Moderate Disability (Barthel Index 41–99 points); ③ Severe Disability (Barthel Index ≤ 40 points). This classification method is widely adopted in older adult care research both domestically and internationally, demonstrating good validity and reliability.

### 2.2 Discrete choice experiment design

Discrete choice experiment is a quantitative method that is usually used to evaluate people's preferences and values for different choices (15). This method is widely used in many fields such as market research, health economics, and environmental science. Its basic idea is to simulate real choice situations and observe the decision-making process of individuals when facing different choices, so as to infer their preferences for various attributes (such as price, quality, service, etc.). In this study, we used this method to conduct a preference analysis on the care needs of older adults with different degrees of disability. The survey questionnaire consists of two parts, namely the basic information of the respondents and the care service preference selection.

#### 2.2.1 Calculation of sample size

Sample size is calculated according to the thumb rule of Johnson and Orme (13, 14). The minimum sample size of a discrete choice experiment should satisfy *N* > 500/t^*^a, where *N* is the sample size, 500 is a fixed variable, c is the maximum number of attribute levels in the experimental design, t is the number of options included in the discrete choice experiment, and a is the number of options for the selected option. In this study, c = 3, *t* = 5, a = 2, and the minimum sample size is calculated to be 150. A total of 600 questionnaires were distributed in this survey, and 579 valid questionnaires were collected.

#### 2.2.2 Discrete choice experiment attribute and level setting

This study determined the attribute pool of the experimental design through literature review (16–18) and expert consultation, and initially focused on the following 10 attributes, namely care service content, care service model, service provider, service quality, service standardization, service price, payment method, feedback mechanism, service flexibility, service accessibility, etc. The final attributes were determined through two rounds of Delphi expert consultation. The expert panel consisted of 15 members, including geriatricians (*n* = 4), health policy researchers (*n* = 4), community health service center managers (*n* = 3), nursing home administrators (*n* = 2), and long-term care insurance policymakers (*n* = 2). The inclusion criteria for experts were: a minimum of 5 years of work experience in the field of older adult health or long-term care, along with having published relevant research or participated in policy investigations. The consensus criteria were defined as follows: ① a mean score for attribute importance ≥4.0 (on a 5-point Likert scale); ② a coefficient of variation (CV) < 0.3; and ③ over 80% of experts agreeing on the inclusion of the attribute. After the two rounds of Delphi expert consultation, the attributes and levels were determined, and finally five attributes were selected, including care service content, service mode, service provider, service price, and payment method. As for service content, it is not easy to quantify, so it is divided into three levels: basic package, upgraded package, and personalized package. The definition of specific attribute content and level is shown in Table 1.

**Table 1:** Discrete choice experiment properties and level settings.

**Factors affecting care services**	**Level (standard)**	**Definition**
Care service content	Life care	Basic package: Life care
	Life care + spiritual care	Upgrade package: Life care + spiritual care
	Life care + mental care + medical care services	Personalized package: life care + mental care + professional medical care
Service method	Home	Home care
	Organization	Nursing home
Service provider	A	Volunteer
	B	Trained caregivers
	C	Professional medical staff
Acceptable single fee	Low	30 (yuan)
	Medium	80 (yuan)
	High	200 (yuan)
Care service cost burden	All at own expense	All care is borne by the person receiving care
	Partial reimbursement	Half of the personal responsibility
	Fully insured	Insurance covers, no personal expenses

#### 2.2.3 Discrete choice experimental design

SAS 9.4 software was used to conduct the D-optimal test. According to the level classification and definition of each attribute, 14 choice sets were generated and evenly distributed to two versions of the questionnaire. Each version of the DCE questionnaire contained 7 choice set questions. To ensure accuracy, 1 repeated question was set at the same time, that is, a total of 8 questions. The questionnaire choice set questions are shown in Table 2. The questionnaire content is mainly designed into two parts: basic demographic information and preference measurement (discrete choice experimental questions). The preference measurement includes two hypothetical conditions for the choice of care services for mildly and moderately disabled older adults and severely disabled older adults. The specific questionnaire is shown in Table 2.

**Table 2:** Example of DCE choice set questionnaire.

**Factors affecting care services**	**Option 1**	**Option 2**
Care service content	Life care + spiritual comfort + medical care services	Life care + spiritual comfort
Service method	Nursing home	Home
Service providers	Volunteer	Trained caregivers
Single fee (yuan)	200	30
Care service cost burden	Partial reimbursement	Fully insured
Your choice		

### 2.3 Statistical methods

This study employed quantitative analysis methods. Descriptive statistics were used to conduct frequency analysis of the respondents' socio-demographic characteristics. The core analytical approach was based on data from a discrete choice experiment (DCE).

Interaction terms between disability level and key sociodemographic variables (gender, income, education, marital status, co-residence) were introduced a priori based on theoretical plausibility. Continuous variables were mean-centered before creating interaction terms to mitigate multicollinearity. The variance inflation factor (VIF) for all independent variables was below 3.0, indicating no severe multicollinearity. A *p*-value of < 0.05 was considered statistically significant for all main and interaction effects. Findings with 0.05 ≤ *p* < 0.10 are described as suggestive trends but are not emphasized for inferential conclusions.

Both conditional logit and mixed logit models were applied to perform regression analysis on data that passed consistency checks. Both conditional logit (CL) and mixed logit (MXL) models were estimated. While the MXL model can account for unobserved preference heterogeneity, the CL model was selected as the primary specification for three reasons: First, the AIC and BIC values for the CL model were consistently lower across all disability subgroups, indicating superior overall fit. Second, the primary research objective focused on estimating population-average preferences rather than modeling the distribution of taste variations. Third, with the inclusion of extensive interaction terms to capture observed heterogeneity, the CL model provided more parsimonious and stable estimates. This study employed both conditional logit and mixed logit models for regression analysis. Model fit was assessed using the Akaike Information Criterion (AIC) and the Bayesian Information Criterion (BIC). The results demonstrated that the conditional logit model yielded lower AIC values (self-care group: 1,917.73; moderate disability group: 2,111.92; severe disability group: 1,224.23) than the mixed logit model (self-care group: 1,925.41; moderate disability group: 2,120.18; severe disability group: 1,232.87). A consistent trend was observed for the BIC values. In accordance with the principle that lower AIC/BIC values indicate better model fit, the conditional logit model was selected as the primary analytical tool. The direction of influence, statistical significance, and relative importance of each attribute were determined by estimating their respective coefficients (beta coefficients, β-values).

Analysis of Willingness-to-Pay. When a monetary attribute is included in the study, the monetary value that respondents place on non-monetary attributes, known as Willingness-to-Pay (WTP), can be assessed by calculating the ratio of the regression coefficients for the non-monetary and monetary attributes (19). This study calculated the monetary value (WTP) of care services for older adult individuals with different disability levels to determine their willingness-to-pay for these services. Based on the calculation of regression coefficients (β), the monetary value for each attribute level was estimated by comparing the β coefficient of the monetary attribute (i.e., acceptable single service fee) with the β coefficients of non-monetary attributes (e.g., care service content, i.e., service package). A positive value indicates the amount respondents are willing to pay to improve a specific attribute level, while a negative value signifies the compensation (subsidy) required for respondents to accept a particular attribute level. The calculation formula is as follows:


WTP=−(β_attribute/β_cost)

Using this formula, the willingness-to-pay for different attribute levels among older adult individuals with varying degrees of disability was derived. On this basis, preferences were converted into economic values by calculating the WTP. Finally, the Akaike Information Criterion (AIC) and Bayesian Information Criterion (BIC) were used to evaluate the model's goodness-of-fit. All the aforementioned statistical analyses were performed using multiple statistical software packages.

## 3 Research results

### 3.1 Basic situation

This study analyzed a total of 579 functionally impaired urban older adult individuals. The sample was predominantly composed of younger seniors aged 60–69 (49.6%), with males accounting for 53.5%. The distribution of disability levels revealed that self-caring older adult comprised 37.7%, moderately disabled 39.9%, and severely disabled 22.5%. Regarding economic status, 67.4% of respondents reported a monthly income below 3,000 CNY, and 42.1% had no health insurance coverage. Educational attainment was generally low, with 77.5% possessing junior high school education or below. Marital status was predominantly married (70.5%), though 16.4% were widowed. The basic characteristics are as follows Table 3.

**Table 3:** Basic information table of samples (*n* = 579).

**Classification**	**No. of examples**	**proportion (%)**	**Classification**	**No. of examples**	**Proportion (%)**
Gender			Disability level		
Male	310	53.5	Self care	218	37.7
Female	269	46.5	Mild to moderate disability	231	39.9
Age			severe disability	130	22.5
60-69 years old	287	49.6	Marital status		
70-79	147	25.4	Married	408	70.5
80-89	88	15.2	Widowed	95	16.4
More than 90	57	9.8	Divorced	44	7.6
Pre retirement career			Unmarried	32	5.5
Cadre	72	12.4			
Worker	126	21.8	Income level (yuan/month)		
Individual	83	14.3	None	89	15.4
Farmer	201	34.7	< 1,000	154	26.6
Soldier	95	16.4	1,000–3,000	147	25.4
No. of children			3,001–5,000	71	12.3
0	28	4.8	5,001–8,000	65	11.2
1	142	24.5	>8,000	53	9.2
2	138	23.8			
3	158	27.3	Medical insurance situation		
≥4	60	10.4	Urban employee medical insurance	87	15.0
Education level			Urban residents medical insurance	134	23.1
Elementary school and below	186	32.1	Publicly funded medical care (savings )	51	8.8
Junior high school	263	45.4	Commercial insurance	24	4.1
High school or technical secondary school	88	15.2	No medical insurance	244	42.1
University and above	42	7.3	Other	39	6.7

### 3.2 Care service preferences of older adults with different degrees of disability

The DCE results revealed that for the self-caring older adults, all attribute levels were statistically significant (*P* < 0.05) except for the personalized care package and full insurance coverage for care service costs. The basic characteristics are as follows Table 4. The basic characteristics are as follows Table 4. The utility weights were as follows, in descending order: care provided by trained caregivers (β = −0.617), partial reimbursement of care service costs (β = 0.476), institutional care (β = 0.213), care provided by professional medical staff (β = −0.194), upgraded care package (β = 0.175), and acceptable single service fee (β = −0.002).

**Table 4:** Preference analysis of factors affecting care services for older adults with different degrees of disability (conditional logit) *n* = 579.

**Attributes and levels**	**Self care**			**Mild to moderate disability**			**severe disability**		
	β	**SE**	**95%CI**	β	**SE**	**95%CI**	β	**SE**	**95%CI**
**Care service content (ref:basic package)**									
Upgrade package	0.175^*^	0.069	(0.038, 0.311)	0,164^*^	0.070	(0.027, 0.300	0.075	0.088	(−0.097, 0.247)
Personalized package	−0.193	0.100	(−0.388, 0.003)	−0.023	0.080	(−0.133, 0.179)	0.053	0.108	(−0.158, 0.265)
**Service method (ref:home)**									
Organization	0.213^***^	0.056	(0.103, 0.324)	0.173^*^	0.054	(0.067, 0.279)	0.153^*^	0.069	(0.018, 0.288)
**Quality of service (Service provider) (ref: A volunteer)**									
B caregiver	−0.617^***^	0.075	(−0.764, −0.469)	−0.559^***^	0.074	(−0.703, −0.414)	−0.331^***^	0.092	(−0.512, −0.151)
C professional medical staff	−0.194^*^	0.096	(−0.381,−0.007)	−0.256^**^	0.082	(−0.417, −0.095)	0.025	0.110	(−0.192, 0.241)
**Cost of care services (ref:All at your own expense)**									
Partial reimbursement	0.476^***^	0.080	(0.319, 0.633)	0.329^***^	0.079	(0.178, 0.479)	0.308^**^	0.098	(0.116, 0.500)
Fully insured	−0.081	0.077	(−0.231, 0.069)	−0.030	0.076	(−0.178, 0.118)	0.163	0.097	(−0.027, 0.354)
Acceptable single fee	−0.002^***^	0.000	(−0.003, −0.001)	−0.001^*^	0.000	(−0.002,−0.000)	−0.000	0.001	(−0.001, 0.001)
Log likelihood	218	231	130						
Sample size	3,054	3,234	1,820						
Observations	−958.863	−1,055.958	−612.113						

β Beta Coefcient, SE Standard error, CI Confdence interval.

^*^p < 0.05,^**^p < 0.01,^***^p < 0.001.

For the moderately disabled older adults, all attributes were statistically significant (*P* < 0.05) except for the personalized care package and full insurance coverage. The utility weights were as follows: care provided by trained caregivers (β = −0.559), partial reimbursement of care service costs (β = 0.329), care provided by professional medical staff (β = −0.256), institutional care (β = 0.173), upgraded care package (β = 0.164), and acceptable single service fee (β = −0.001).

For the severely disabled older adults, among all attributes, only institutional care, care provided by trained caregivers, and partial reimbursement of care service costs were statistically significant (*P* < 0.05). The utility weights were as follows: care provided by trained caregivers (β = −0.331), partial reimbursement of care service costs (β = 0.308), and institutional care (β = 0.153).

### 3.3 Analysis of Preference Heterogeneity

**Figure 1:**
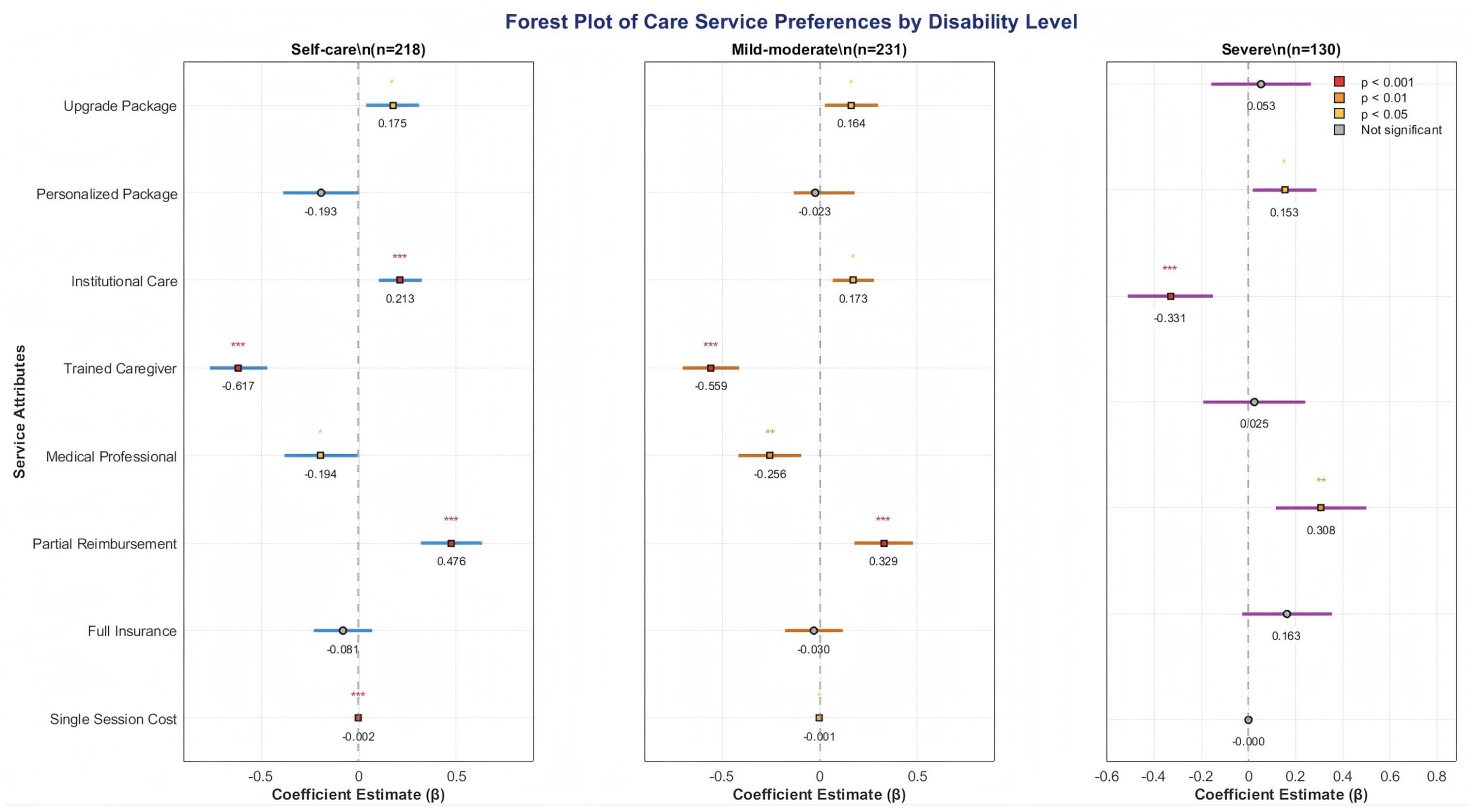
forest plot of care service preferences among older adult with different disability levels (95% CI). *p < 0.05, **p < 0.01, ***p < 0.001.

A clear gradient in preferences was observed across disability levels. While all groups significantly dispreferred trained caregivers relative to volunteers, the magnitude of this negative preference diminished with increasing disability severity (Self-care: β = −0.617; Moderate: β = −0.559; Severe: β = −0.331). Conversely, the preference for institutional care over home-based care intensified with disability level, becoming statistically significant only in the severe disability group. Show as Figure 1 and Table 4.

#### 3.3.1 Care preferences of self-caring older adults

The self-care older adult group exhibited significant positive preferences for the “Upgrade Package” (β = 0.175, 95%CI: 0.04–0.31) and “Partial Reimbursement” (β = 0.476, 95%CI: 0.32–0.63), while showing the strongest negative utility toward “Trained Caregivers” (β = −0.617, 95%CI: −0.76–0.47). Interaction analysis further revealed a negative interaction between female gender and the caregiver coefficient (interaction β = −0.124, *p* < 0.05), suggesting that self-care females, potentially due to their familial caregiving roles, exhibit stronger rejection of external caregivers. A positive interaction was found between higher education levels and the upgrade package (interaction β = +0.112, *p* < 0.01), indicating individuals with higher education demonstrate greater recognition of the value of services like spiritual comfort, shown in Table 5.

**Table 5:** Heterogeneity in care preferences of self-caring older adults.

**Attribute**	**Main Preference**	**Source of Heterogeneity**
Service method	Prefer Home-based (β = 0.213)	Female vs. Male: Females more averse to Institutional care (interaction β = −0.124^*^)
Service provider	Strongly Averse to Trained Caregiver(β = −0.617)	Higher Education: Stronger aversion to Trained Caregiver (interaction β = −0.201^**^)
Cost of care services	Prefer Partial Reimbursement (β = 0.476)	Low Income: Stronger negative preference for “Full Insurance” (interaction β = −0.178^*^)

#### 3.3.2 Care preferences of moderately disabled older adults

The moderately disabled group still significantly preferred the “Upgrade Package” (β = 0.164) and “Partial Reimbursement” (β = 0.329), but their acceptance of “Institutional Care” had slightly increased (β = 0.173). Overlapping confidence intervals in the forest plot indicated that the difference from the self-care group was not statistically significant, suggesting this group is in a transition period for preferences. Interaction results found that those co-residing with children were willing to pay 58 CNY more for the upgrade package (*p* < 0.1), and individuals with rural household registration showed a negative interaction with institutional care (interaction β = −0.211, *p* < 0.01), reflecting geo-cultural resistance. This group is willing to receive higher-level services in a familiar environment but remains wary of “leaving home,” shown in Table 6.

**Table 6:** Heterogeneity in care preferences of moderately disabled older adults.

**Attribute**	**Main preference**	**Source of heterogeneity**
Care service content	Prefer Upgrade Package (β = 0.164)	Co-residing with Children: Higher WTP for Upgrade Package (+58.06 CNY^*^)
Service method	Prefer Home-based(β = 0.173)	Rural Hukou: More averse to Institutional care (interaction β = −0.211^**^)
Cost of care services	Partial Reimbursement(β = 0.329)	Higher tolerance for “Full Self-payment” (interaction β = +0.089^*^)

#### 3.3.3 Care preferences of severely disabled older adults

In the severely disabled group, only “Institutional Care” (β = 0.153, CI: 0.02–0.29) and “Partial Reimbursement” (β = 0.308, CI: 0.12–0.50) were significantly positive. Preference regarding “Trained Caregivers” remained negative but the magnitude was reduced (β = −0.331), indicating a shift in focus toward medical care and financial risk sharing. Interaction analysis indicated that individuals without a spouse were willing to pay 314 CNY more for institutional care (*p* < 0.01), and those with a history of chronic disease showed a positive interaction with professional medical care (interaction β = +0.198, *p* < 0.05), suggesting that lack of family support and health complexity jointly increase the demand for institutional care. However, their payment ability is generally low (< 3,000 CNY/month), creating a “high willingness - low ability” trap, shown in Table 7.

**Table 7:** Heterogeneity in care preferences of severely disabled older adults.

**Attribute**	**Main preference**	**Source of heterogeneity**
Service provider	Not averse to Professional Medical Staff (β = 0.025, NS)	Without Spouse: Higher WTP for Institutional care (+314.2 CNY^**^)
Service method	Prefer Institutional Care (β = 0.153)	Chronic Disease History: Significantly prefer Professional Medical Staff (interaction β = +0.198^*^)
Cost of care services	Prefer Partial Reimbursement(β = 0.308)	Pension >3000 CNY: No significant preference for “Full Insurance” (interaction β = −0.089, NS)

#### 3.3.4 Stratified heterogeneity interaction effects (mixed logit model)

Integrated analysis of forest plots and interaction models revealed dual differences in “utility strength” and “sign direction” for the same attribute across different disability levels: Firstly, the negative utility of “Trained Caregiver” decreased as disability severity increased (Self-care: −0.617 → Severe: −0.331), but females and individuals with lower education consistently exhibited stronger rejection across all levels. Secondly, the positive utility of “Institutional Care” shifted from non-significant in the mild group to significant in the severe group, amplified by interactions with being unmarried and having low income. Thirdly, “Partial Reimbursement” was positively significant across all levels, but individuals with pensions >3,000 CNY showed no incremental preference for “Full Insurance,” indicating that “co-payment” aligns better with the older adult's mindset than “full coverage.” The results demonstrate that disability level is the primary factor determining the “structure” of preferences, while individual characteristics like gender, marital status, and income determine the “strength,” supporting the development of a dual-dimensional targeting strategy based on “disability level - individual characteristic,” shown in Table 8.

**Table 8:** Stratified heterogeneity interaction effects in older adult care preferences.

**Disability level**	**Attribute Level**	**Main Effect β**	**Interaction Variable**	**Interaction Coef**.
Self care	Trained Caregiver	−0.617^***^	Female	−0.124^*^
Mild to moderate disability	Upgrade Package	0.164^**^	Co-residing with Children	0.089^*^
Severe disability	Institutional Care	0.153^*^	Without Spouse	0.211^**^

^*^p < 0.05, ^**^p < 0.01, ^***^p < 0.001.

### 3.4 Willingness to pay for each attribute under the assumption of different degrees of functional limitation of the older adult

The results of the willingness-to-pay (WTP) analysis, as shown in Table 9, indicate that regarding care service content, the WTP for the upgrade package was statistically significant (*P* < 0.05) for both the self-caring and moderately disabled older adults groups. The moderately disabled group exhibited a higher WTP for the upgrade package, at 149.372 CNY. If the care service content were switched from the “basic package” to the “upgrade package,” moderately disabled older adults individuals were willing to pay 58.059 CNY more than their self-caring counterparts. No statistically significant WTP (*P* > 0.05) for the personalized package was found across all three disability level groups.

**Table 9:** Willingness to pay for various attributes of care services for older adults with different degrees of disability (yuan).

**Attributes and levels**	**Self care**		**Mild to moderate disability**		**Severe disability**	
	**WTP/yuan**	**95%CI**	**WTP/yuan**	**95%CI**	**WTP/yuan**	**95%CI**
**Care service content (ref:basic package)**						
Upgrade package	91.313	(5.355, 177.271)	149.372	(−15.172, 313.915)	441.418	(−2,704.578, 3,587.415)
Personalized package	−100.734	(−207.936, 6.468)	20.794	(−121.726, 163.315)	314.576	(−2,225.907, 2,855.059)
**Service method (ref:home)**						
Organization	111.590	(34.035, 189.146)	158.080	(−3.284, 319.444)	899.563	(−5,297.991, 7,097.116)
**Quality of service (service provider) (ref:A volunteer)**						
B caregiver	−322.473	(−496.695, −148.251)	−509.231	(−942.024, −76.439)	−1949.401	(−15,273.697, 11,374.894 )
C professional medical staff	−101.491	(−205.998, 3.016)	−233.586	(−480.602, 13.431)	144.118	(−1483.086, 1,771.323)
**Cost of care services (ref:All at your own expense)**						
Partial reimbursement	249.046	(94.407, 403.685)	299.625	(39.184, 560.066)	1811.032	(−10,568.945, 14,191.009)
Fully insured	−42.336	(−127.032, 42.361)	−27.298	(−166.140, 111.544)	961.155	(−5,663.640, 7,585.950)

#### 3.4.1 Service delivery mode

The severely disabled older adults exhibited the highest willingness-to-pay (WTP) for institutional care, at 899.563 CNY. This was followed by the moderately disabled older adults (WTP = 158.080 CNY) and the self-caring older adults (WTP = 111.590 CNY). If the service delivery mode were switched from “home-based” to “institutional” care, severely disabled older adults individuals were willing to pay 741.483 CNY more than their self-caring counterparts.

#### 3.4.2 Service provider

Compared to services provided by volunteers, older adult individuals across all disability levels showed a dispreference for services delivered by trained caregivers or professional medical staff, as indicated by their negative WTP values. A switch from services provided by “volunteers” to those provided by “trained caregivers” would require compensation of 322.473 CNY for self-caring older adults. The required compensation for the severely disabled older adults was six times higher (1,949.401 CNY). Similarly, a switch from “volunteer” services to those provided by “professional medical staff” would require compensation of 101.491 CNY for the self-caring group and 233.586 CNY for the moderately disabled group. The WTP of the severely disabled older adults for professional medical staff services was not statistically significant (*P* > 0.05).

#### 3.4.3 Cost burden

The severely disabled older adults showed the highest WTP for partial reimbursement, at 1,811.032 CNY, followed by the moderately disabled group (WTP = 299.625 CNY) and the self-caring group (WTP = 249.046 CNY). If the payment method were switched from “full self-payment” to “partial reimbursement,” severely disabled older adults individuals were willing to pay 1,561.986 CNY more than those in the self-caring group. The WTP of the severely disabled older adults for full insurance coverage was not statistically significant (*P* > 0.05). Conversely, the self-caring and moderately disabled groups exhibited negative WTP for full insurance, meaning they would require compensation of 42.336 CNY and 27.298 CNY, respectively, to accept a switch from “full self-payment” to “full insurance.”

#### 3.4.4 Heterogeneity in willingness-to-pay

WTP was calculated using the ratio of the forest plot beta coefficients to the cost attribute coefficient, combined with interaction term analysis. The results, summarized in Table 10, show that: (1) Self-caring individuals were willing to pay 91 CNY for the upgrade package, but this WTP increased to 149 CNY among the highly educated subgroup; (2) The WTP for institutional care was 158 CNY among the moderately disabled, but decreased by 41 CNY for those with rural household registration; (3) The severely disabled group had a very high WTP for institutional care (899 CNY), yet the low-income subsample within this group comprised only 32%, highlighting the most pronounced mismatch between high need and low payment capacity. Regarding the cost burden, the severely disabled group's WTP for partial reimbursement reached 1,811 CNY, which is 7.3 times that of the self-caring group, while showing no significant incremental WTP for “full insurance.” This reveals their greater concern for “risk sharing” rather than “free services.”

**Table 10:** Willingness-to-pay stratified by disability level and income (Unit: CNY).

**Income group**	**Self-Care - WTP (Institutional)**	**Moderate Disability - WTP (Upgrade Pkg)**	**Severe Disability - WTP (Institutional)**
Low Income	111	149	899
Middle Income	98	132	756
High Income	76	89	621

The wide confidence intervals for WTP estimates, particularly in the severe disability group (e.g., institutional care: 95% CI −5,297.99 to 7,097.12 CNY), indicate considerable estimation uncertainty. This is likely attributable to the smaller subsample size (*n* = 130), substantial preference heterogeneity within the group, and the high sensitivity of WTP calculations to the denominator (cost coefficient). Consequently, the point estimates for WTP in the severe disability group should be interpreted with caution as indicative of a high valuation tendency rather than precise monetary amounts. Future studies with larger samples are needed to obtain more stable WTP estimates for this vulnerable subgroup.

## 4 Discussion and suggestions

### 4.1 The impact of disability level on heterogeneity of service preferences

This study revealed the significant moderating effect of disability level on the preference for older adult care services through a discrete choice experiment. People with mild to moderate disabilities are more inclined to choose home-based care and upgraded service packages, while showing significant rejection of volunteer services and self-payment methods. This result is consistent with the theory of “community-embedded older adult care”(20) mentioned by Wang Ji tong, i.e, older adults with mild to moderate disabilities still retain a certain degree of autonomy, and home services can meet their needs for environmental familiarity and social participation. At the same time, upgraded service packages (such as spiritual comfort) can alleviate their psychological pressure caused by functional limitations (21). However, the negative preference for volunteer services reflects the implicit requirement of this group for the professionalism of services. Although their functional limitations are relatively low, they still expect to obtain standardized care support rather than relying on non-professional volunteers. In contrast, the strong preference of the severely disabled group for professional medical teams and service upgrade packages highlights their high health risks and strong dependence. This group often requires complex medical care (such as wound treatment, respiratory support, and pipeline maintenance), and the skill guarantee of professional medical staff has become a core demand. It is worth noting that despite severe disability, this group of people also prefer home services, but their rejection of institutional services is lower than that of the mild and moderate groups, suggesting that when physical function is severely limited, service quality takes precedence over environmental comfort. This finding challenges the traditional simple assumption that “home is better than institutions” and suggests that policy design needs to dynamically adjust the service model in combination with the degree of disability. Constructing a hierarchical assessment system: Establishing a dynamic assessment mechanism based on ADL scores, providing modular services of “home + community support” (22) for people with mild and moderate disabilities, such as embedded psychological counseling; building an integrated care network led by “professional medical care” for people with severe disabilities, and promoting the sinking of resources of tertiary hospitals through medical alliances (23).

Differentiated Service Models: The strong preference for home-based care among the self-care and moderately disabled groups supports policies that strengthen “Aging in Place” through community-based service upgrades (24). In contrast, the significant preference for institutional care among the severely disabled calls for targeted investment in professional nursing facilities.

### 4.2 Economic fairness and institutional optimization path of differences in willingness to pay

WTP analysis further reveals the impact of disability level on economic decision-making. The mild and moderately disabled group is willing to pay 159 yuan for the upgraded service package, while the severely disabled group is willing to pay as high as 362 yuan. This difference may be due to two aspects: first, the medical expenditure of severely disabled people is more rigid, and the family economic burden is heavier, resulting in their lower ability to pay for high-quality services (25); second, the resistance of the mild and moderate groups to out-of-pocket payment reflects their higher economic sensitivity, which may be related to limited pension or insufficient family support (26). It is noteworthy that the high willingness-to-pay for institutional care and partial reimbursement among the severely disabled group (899.56 CNY and 1,811.03 CNY, respectively) stands in sharp contrast to their generally low income levels (67.4% of respondents in this study reported monthly incomes < 3,000 CNY), highlighting a structural disparity between high care needs and low payment capacity. This phenomenon suggests that relying solely on out-of-pocket payments would place families with severely disabled members at risk of poverty due to care costs. Therefore, policy interventions must prioritize enhancing the foundational role of public financing and long-term care insurance, establishing a disability-level-linked cascading subsidy mechanism to ensure a balance between service accessibility and financial affordability.

Tiered Reimbursement in LTCI: The consistent, strong positive WTP for ‘partial reimbursement' across all groups, coupled with indifference or aversion to ‘full insurance,' suggests that long-term care insurance (LTCI) schemes should prioritize designing co-payment mechanisms rather than seeking full coverage (27). Reimbursement rates could be tiered based on disability level.

### 4.3 Contradictions and breakthrough directions in the professionalization of service providers

Research shows that both mild and moderate and severe disabled groups show significant rejection of volunteer services, which is contrary to the conclusions of some studies advocating “voluntary older adult care” (28). In-depth analysis found that the unprofessionalism and instability of volunteer services are the main contradictions. For example, it is difficult for volunteers to perform professional nursing operations (such as catheter maintenance), and the service time is fragmented, which cannot meet the continuous needs of disabled older adults (29). In addition, in terms of cultural concepts, the older adult may regard accepting volunteer help as “externalization of family responsibilities” and have psychological resistance. However, the high preference of the severely disabled group for professional medical staff indicates that their core demand lies in service quality rather than service form. This is consistent with the trend of “professional home medical care” in developed countries (30).

Workforce Professionalization: The pervasive rejection of both volunteer and trained caregiver services underscores a critical demand for quality and professionalism (31). Policy should focus on standardizing training, certification, and career pathways for care workers to build trust and meet the explicit demand for professional medical staff.”

## 5 Innovation and insufficiency

This study has made breakthroughs in methodology, theory and practice. Methodologically, the discrete choice experiment (DCE) was combined with the mixed logit model for the first time to quantitatively analyze the heterogeneous impact of disability on care service preferences, overcoming the limitation of the traditional logit model that ignores individual differences, and converting preferences into economic value through willingness to pay (WTP) analysis, providing a new tool for policy cost-effectiveness evaluation. In theory, the dynamic regulatory effect of disability on service selection was revealed, and a hierarchical adaptation framework of “mild, moderate, intensive autonomy, severe, intensive professionalism” was proposed.

The study sample was primarily drawn from Nanjing, a representative city in the economically developed eastern region of China. The city has a relatively well-established older adult care system and exhibits considerable heterogeneity in its older adult population. Consequently, the findings may offer valuable insights for similar urban contexts (e.g., Hangzhou, Suzhou, Shanghai). However, given China's vast geographical expanse and notable urban-rural disparities, significant differences exist in service accessibility, payment capacity, and cultural beliefs and preferences in rural areas. Therefore, the generalizability of our findings to rural settings may be limited. Future research should involve multi-center, multi-regional studies to validate the regional heterogeneity of preferences.

## Data Availability

The original contributions presented in the study are included in the article/supplementary material, further inquiries can be directed to the corresponding author.
